# Effect of Kinesio^®^ Taping on Ankle Complex Motion and Stiffness and Jump Landing Time to Stabilization in Female Ballet Dancers

**DOI:** 10.3390/jfmk4020019

**Published:** 2019-04-08

**Authors:** Aline E. Botsis, Neil A. Schwarz, Megan E. Harper, Wei Liu, Collin A. Rooney, Larry R. Gurchiek, John E. Kovaleski

**Affiliations:** 1 Department of Health, Kinesiology, and Sport, University of South Alabama, Mobile, AL 36688, USA; 2 Department of Biomedical Affairs and Research, VCOM-Auburn University, Auburn, AL 36832, USA; 3 Ipsos NA, 300 Corporate Pointe, Culver City, CA 90230, USA

**Keywords:** ankle arthrometry, ankle sprain, ballet dancers, Kinesio tape, time to stabilization

## Abstract

Ankle sprain is the most commonly diagnosed injury experienced by ballet dancers with few studies investigating preventive support measures such as Kinesio taping. The need exists to examine the mechanical support characteristics of Kinesio taping and effect of application on ankle motion and performance. This may be important to understanding the mechanical mechanisms attributed to Kinesio ankle taping and justify its use in the prevention and treatment of jump landing injuries in ballet dancers. This study compared Kinesio taping with and without tension and no tape (control) on active and passive measures of ankle complex motion in healthy ballet dancers. A secondary objective was to examine the effect of Kinesio taping on balance using time to stabilization. Participants performed three ballet jumps with single-leg landings on a force plate across three ankle support conditions consisting of Kinesio taping, sham-Kinesio taping, and no tape. Sagittal and frontal plane motion and load-displacement of the ankle complex for each support condition were obtained using an ankle arthrometer. Kinesio taping with tension significantly restricted inversion-eversion rotation and increased inversion stiffness of the ankle complex (*p* < 0.05). No significant differences were found among the three ankle support conditions for jump landing time to stabilization (*p* > 0.05). Arthrometric results indicate Kinesio taping significantly restricted ankle complex motion in the frontal plane that is associated with lateral ankle sprain. Objective information on the nature of Kinesio taping support can assist sports medicine practitioners when recommending ankle support to athletes.

## 1. Introduction

Classical ballet movements and jump landings require ankle range of motion and stability when balancing in extreme single-leg stance postures [1,2]. Sufficient ankle plantarflexion is vital to achieving full pointe and postural control is important to technical execution and the elegance of movement associated with ballet [3]. The physical demands of ballet, often paired with strenuous training and performance schedules, result in lateral ankle sprain being the most common ankle injury among female ballet dancers [4,5,6]. 

Kinesio taping, a recently developed and popular method of taping is considered potentially beneficial to use with ballet dancers [7]. Kinesio taping is purported to allow normal ankle plantar- dorsiflexion range of motion while reducing accessory anterior-posterior translation of the ankle complex and is purported to support and stabilize the ankle joint through enhanced proprioception, increased muscle activation, and/or alteration of perception of exercise [8,9,10]. Methodological differences in ankle tape application include variations in tape application procedures, such as the nature of skin preparation, the amount of tension generated within tape strips as they are adhered to the skin, and the orientation of tape strips in relation to anatomic structures. While traditional athletic tape is typically applied solely around the ankle joint using stirrups, a figure-of-eight configuration, and heel-locks, Kinesio tape can be applied over and around muscles to provide support [11]. To our knowledge, no studies have been reported showing Kinesio tape acts as a mechanical constraint to inversion-eversion motion within the frontal plane. In addition, limited information exists on joint elasticity (stiffness) variables of the ankle complex with Kinesio taping [10]. 

Meta-analysis of previous Kinesio taping investigations involving the lower extremity and ankle functional performance show that the Star Excursion Balance Test and vertical jump results for Kinesio taping were superior to placebo taping and tension-free taping and had no effect on range of motion [12]. Given that plantarflexion and inversion rotation of the ankle complex are associated with lateral ankle sprain, quantifying the effect of Kinesio taping on the ankle complex and jump landing postural control is relevant in the dance population [11,13,14]. Jump landing forces at ground contact can be used to elucidate balance-control strategies such as time to stabilization (TTS). TTS calculates the time (in seconds) it takes post landing for an individual’s ground reaction forces to stabilize to the level of normal quiet stance. An ability to stabilize quickly is generally considered as a positive or protective trait. The effect of Kinesio taping on time to stabilization during jump landing has not been previously reported in ballet dancers. Research findings indicate a link between ankle injury and subsequent deficits in balance, which is important to the ballet dancer when performing complex movements that require jumping and landing [1,15,16,17].

The need exists to examine the mechanical support characteristics of Kinesio tape and its effect of application on ankle motion and performance. This may be important in identifying and understanding the mechanical support mechanisms attributed to Kinesio ankle taping to justify its use in the prevention and treatment of jump landing injuries. The primary purpose of this study was to compare Kinesio taping with and without tension and no tape (control) on active and passive measures of ankle complex motion in healthy ballet dancers. Additionally, it is important that taping does not hinder performance or the aesthetics of the dance choreography. A secondary objective was to examine the effect of Kinesio taping on balance using TTS in the execution of sauté arabesque, sissonne ouverte de côté, and sissonne ouverte an evant single-leg jump landings. We hypothesized Kinesio tape application with tension would decrease ankle complex anterior-posterior translation and inversion-eversion rotation, increase anterior-posterior and inversion-eversion ankle complex stiffness, and not restrict plantarflexion (PF) and dorsiflexion (DF) range of motion (ROM) when compared to Kinesio tape application without tension and wearing no-tape. Regarding TTS, we hypothesized that Kinesio taping with tension would reduce jump landing time to stabilization when compared to Kinesio tape application without tension and wearing no-tape, but did not expect to find differences between the Kinesio tape application without tension and wearing no-tape.

## 2. Materials and Methods 

### 2.1. Study Design

This study employed a mixed-model, repeated measures design. The independent variables included ankle support condition with three levels (Kinesio taping with tension, Kinesio taping without tension [sham-Kinesio taping], and no-taping) and ballet jump landing with three levels (sauté arabesque, sissonne ouverte de côté, and sissonne ouverte an evant). Dependent variables included passive measures of anterior-posterior and inversion-eversion ankle complex motion and stiffness; active PF and DF ROM, and jump landing time to stabilization.

### 2.2. Participants

12 non-professional female ballet dancers (18.25 ± 2.5 years; height: 160.21 ± 7.89 cm; weight: 65.69 ± 18.59 kg.) with no reported history of ankle or lower leg injury participated. Mean experience dancing en pointe was 5.91 ± 2.6 years. An a priori power analysis showed that in order to detect large-sized effects (i.e., a partial eta-squared (η2) value of 0.14) between conditions with 80% power, at least 12 participants were required. This study was approved by the University of South Alabama Institutional Review Board (IRB: 14-188; August 14, 2018), and all subjects age 18 or over signed informed consent forms. Dancers under 18 years of age signed an assent form, and their parent signed a parental permission form. 

### 2.3. Instrumentation

Testing of ankle complex passive and active motions was conducted using a portable ankle arthrometer (Blue Ray Research Inc., Navarre, FL, USA). Ankle arthrometry is an objective method for assessing non-weight bearing translatory and angular motions of the foot in relation to the leg that result from the combined motions of the talocrural and subtalar joints. The ankle arthrometer has been reported to be highly reliable for examiner intratester reliability (anteroposterior translation: Intraclass Correlation Coefficient [ICC] = 0.98, Standard Error of the Mean [SEM] 0.89 mm and for Inversion-eversion rotation: ICC = 0.91, SEM = 0.98^◦^) and a valid tool for ankle ligamentous stability assessment [18,19]. High validity of measurement has been derived by comparison with concurrent measurement of tibial-calcaneal bone motion in cadaver specimens for sagittal-plane translation (*r* = 0.88) and frontal-plane rotation (*r* = 0.86).

**Figure 1:**
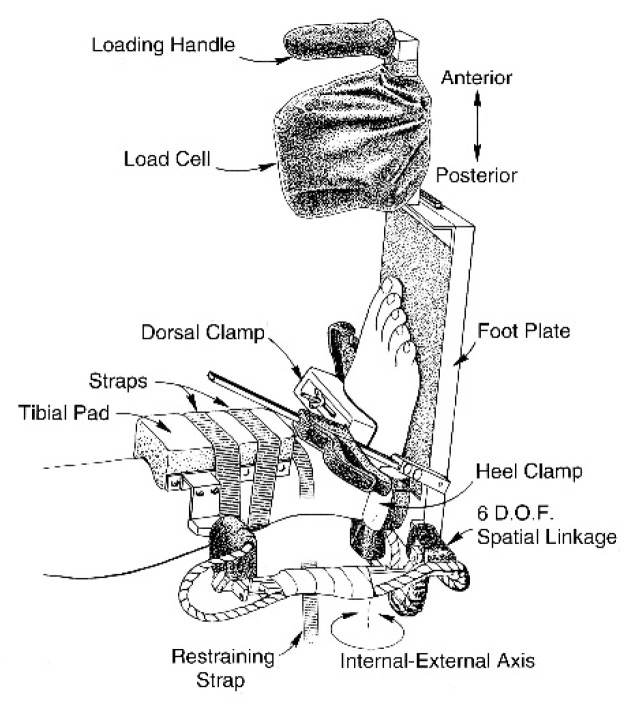
Ankle arthrometer.

As seen in Figure 1, the arthrometer consists of a spatial kinematic linkage, an adjustable plate fixed to the foot, a load-measuring handle attached to the footplate through which the load is applied, and a reference pad attached to the tibia [20]. The spatial kinematic linkage is a six-degrees-of-freedom electrogoniometer that measures applied forces and moments and the resultant translations and rotations of the ankle complex [21]. The arthrometer spatial linkage connects the tibial pad to the footplate and measures the motion of the footplate relative to the tibial pad. When load is applied to the handle attached to the footplate, the spatial linkage uses the electrogoniometer to measure anteroposterior (AP) load displacement and inversion-eversion (I-E) rotation. In addition, the electrogoniometer measured ankle-flexion angle from the plantar surface of the foot relative to the anterior tibia. The resulting AP displacement (millimeters) and I-E rotation (degrees of range of motion) along with the corresponding AP load and I-E torque were recorded. A custom software program written in LabVIEW (National Instruments) was used for collection and reduction of the data.

Jump landing time-to-stabilization was assessed with a force plate (Advanced Mechanical Technologies Inc., Watertown, MA, USA) mounted to a wooden platform. The force platform was calibrated with known loads to the voltage recorded before testing. Kinetic data was collected at 180 Hz, real time displayed, and analyzed using BioAnalysis 3.1 software (Advanced Mechanical Technologies Inc., Watertown, MA, USA).

### 2.4. Data Collection

#### 2.4.1. Taping Procedure

The dominant ankle was determined as the foot on which each dancer landed when performing the sissonne and sauté arabesque jumps. All participants were randomly assigned and participated in each of three support trials: KT (Kinesio taping), ST (sham-Kinesio taping), and NT (no taping).

The dominant ankle was taped for prevention of a lateral ankle sprain in accordance with Kinesio™ Tape guidelines as recommended by the Kinesio Taping Association International. The same taping configuration was applied for both taped supports by the principle investigator. The Kinesio tape application for the KT trial was applied at 90–100% of the tape’s maximum length. The Kinesio tape application for the ST trial was applied in the same configuration but without tension. Prior to application, the foot and ankle were cleaned with alcohol. Each participant was blinded to the type of tape application to reduce the possibility of subject bias. 

**Figure 2:**
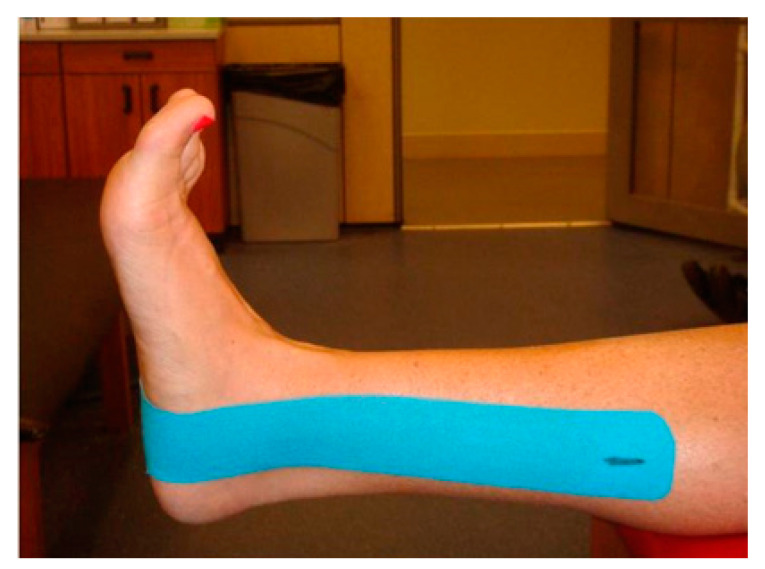
Medial view of the first strip of Kinesio tape.

For the KT and ST trials, three strips of Kinesio tape were applied with the foot held in full dorsiflexion. Anatomical landmarks were used as a guide to apply each tape strip. Figure 2 shows the stirrup strip applied to the plantar surface of the foot just anterior to the calcaneous. The lateral side of the stirrup was stretched over the lateral malleolus, and then the medial side of the stirrup was stretched over the medial malleolus. 

**Figure 3:**
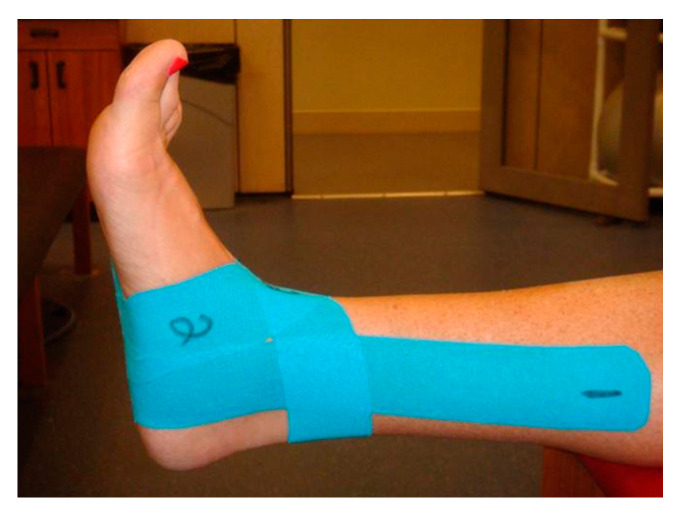
Medial view of the second strip of Kinesio tape.

**Figure 4:**
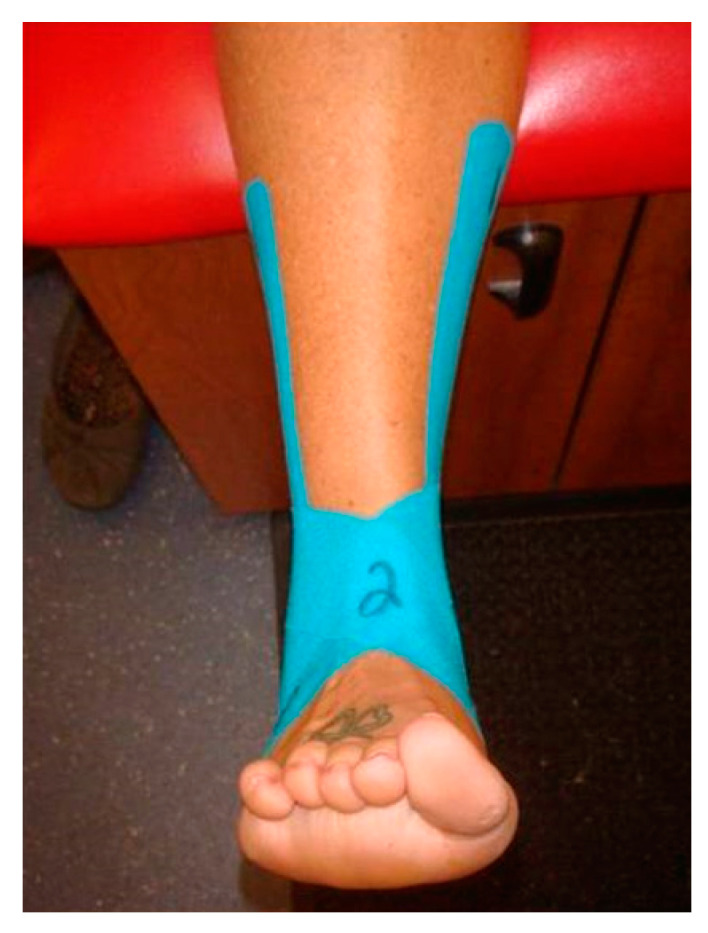
Anterior view of the second strip of Kinesio tape.

Figure 3 and Figure 4 show the figure-of-eight strip, which was applied perpendicular to the Achilles tendon. The lateral side of the strip was stretched over the lateral malleolus and over the dorsal aspect of the foot to close on the medial plantar surface of the foot. The medial side of the strip was stretched over the medial malleolus and over the dorsal aspect of the foot to close on the lateral plantar surface of the foot.

**Figure 5:**
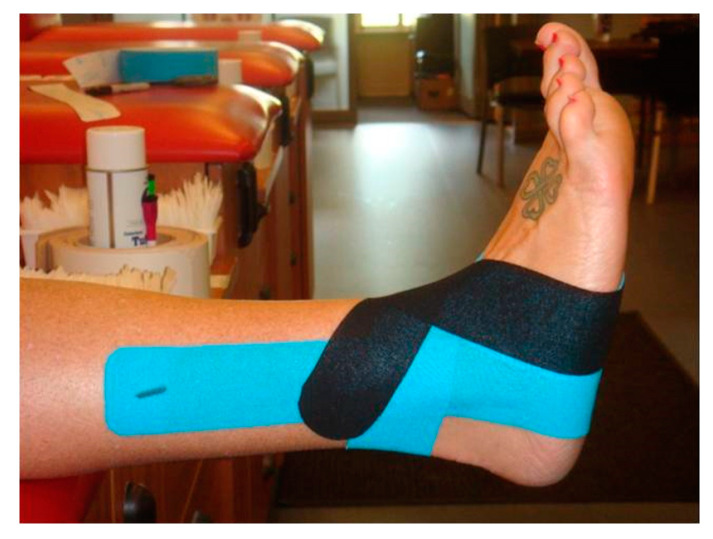
Lateral view of the third strip of Kinesio tape.

**Figure 6:**
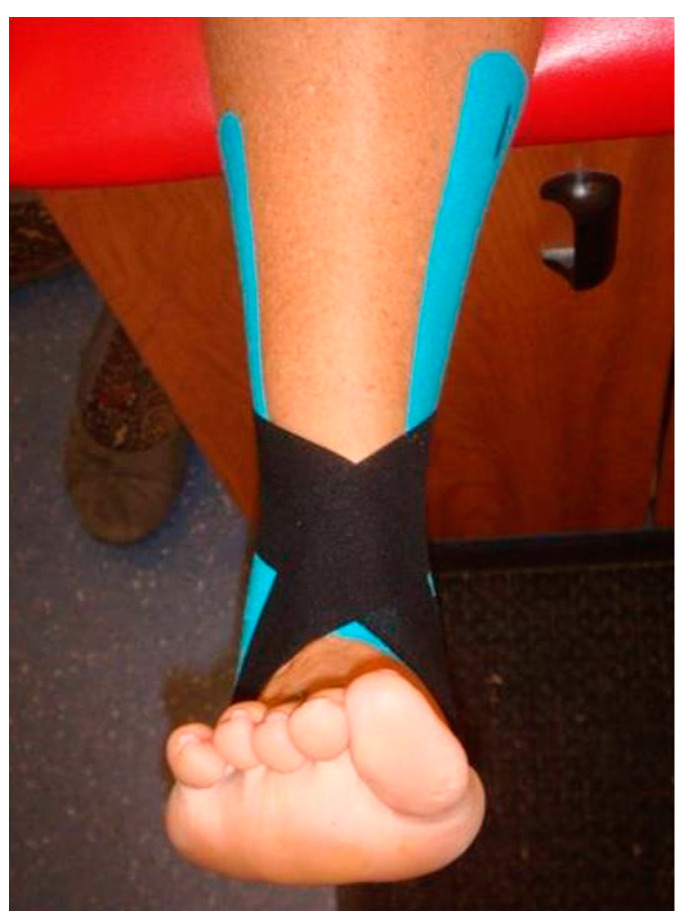
Anterior view of the third strip of Kinesio tape.

The third strip (Figure 5 and Figure 6) was applied first to the plantar surface of the foot anterior to the calcaneous and just anterior to the first strip. The lateral side of the strip was stretched over the dorsal aspect of the foot and over the medial malleolus to close perpendicular to the Achilles tendon. The medial side of the strip was stretched over the dorsal aspect of the foot and over the lateral malleolus to close perpendicular to the Achilles tendon. After application of all strips, the tape was rubbed for 20–30 s to ensure activation and adherence of the tape’s glue.

#### 2.4.2. Ballet Jump Procedures

**Figure 7:**
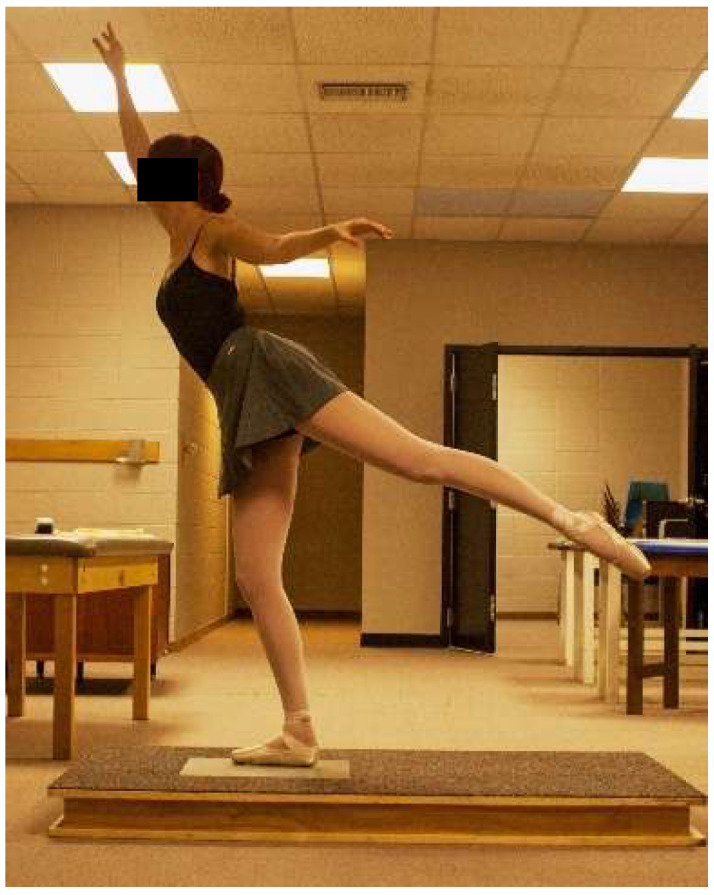
Jump landing in plié on the force plate for sauté arabesque.

**Figure 8:**
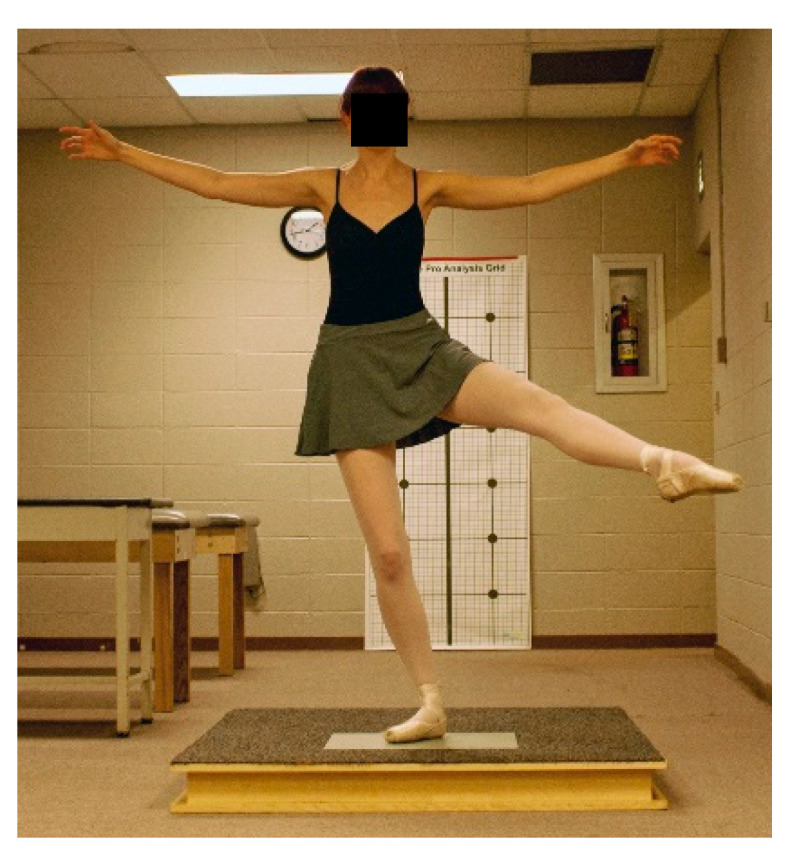
Jumping landing in plié on the force plate for sissonne ouverte de côté.

**Figure 9:**
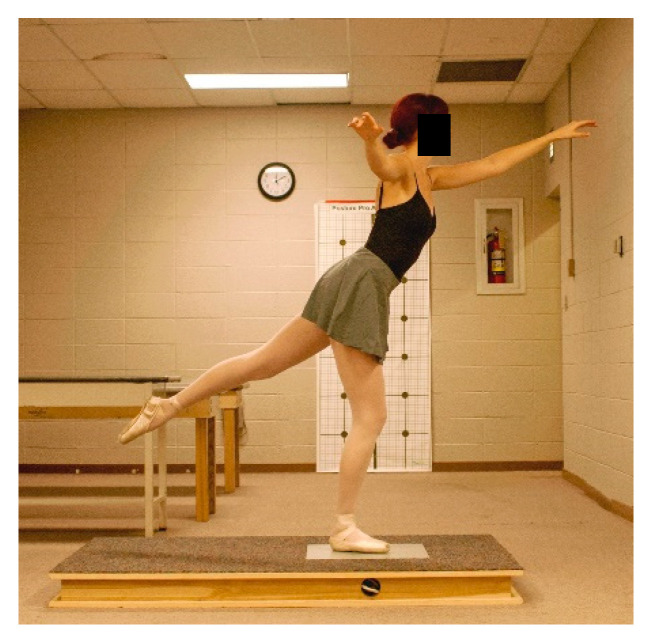
Jumping landing in plié on the force plate for sissonne ouverte an evant.

Participants warmed-up by performing static stretching and dynamic movements and were allowed to practice all jumps prior to data collection. Jump landings resulting in stumbling, touching down with the non-weight bearing leg, or incorrectly executed were repeated. In each ankle support trial, participants were randomly assigned and performed three ballet movements and jumps that ended in a single-leg landing on the dominant leg for a sauté arabesque (Figure 7), sissonne ouverte de côté (Figure 8), and sissonne ouverte (Figure 9). All jumps were performed in pointe shoes. All jumps were performed facing a mirror to provide visual feedback and simulate common training practice [22]. Arm positioning was standardized for each jump. All jumps began off the force plate and ended with a single-leg landing onto the center of the force plate. Upon landing, the participants were instructed to stabilize as quickly as possible and remain motionless for 10 s. 

#### 2.4.3. Ankle Arthrometric Procedures

Our testing procedures replicated previously reported research [10,18,19,20,23]. Ankle motion testing replicated previously reported methods and was performed after the completion of each jump for each support condition. Each participant removed her pointe shoe and was positioned supine on a padded table with the knee supported in 15 to 20 degrees of flexion and the foot positioned off the table [20]. To prevent lower leg movement, a restraining strap attached to the support bars beneath the table was secured and tightened around the distal lower leg approximately 1 cm superior to the malleoli. The examiner secured the arthrometer to the foot by placing the bottom of the foot onto the footplate and adjusting the heel and dorsal clamps. The heel clamp prevented the device from rotating on the calcaneus, while the dorsal clamp secured the foot to the footplate. A pad positioned 5 cm above the ankle malleoli was secured to the tibia. To minimize the variation between the forces applied to the ankle, the arthrometer was oriented in a similar manner on each leg for all tests. The force loads administered by the examiner were applied through the load handle in line with the footplate. 

To measure passive AP displacement and I-E rotation, the foot was positioned at zero AP load, zero I-E moment, and a neutral (0˚) flexion angle, which was the measurement reference position. To record AP displacement, the ankle was loaded with 100 N of anterior and posterior force. Starting at the neutral position, an anterior load was applied initially, followed by a posterior load. For I-E rotation, the ankles were loaded to 4 N·m of inversion and eversion torque. Starting at the neutral position, inversion loading was applied first, followed by eversion loading. The computer monitor was visualized to control the application of force required to obtain the maximum load of 100 N for AP displacement and 4 N·m for I-E rotation [18,19,20].

To measure non-weight-bearing active dorsiflexion (DF) and plantarflexion (PF) the subject maximally plantarflexed and dorsiflexed her foot. The ankle was positioned at neutral (0 degrees of flexion), which was defined as the measurement reference position [18,19,20]. This angle was measured from the plantar surface of the foot relative to the anterior tibia via the tibial reference pad and determined by the six-degrees-of-freedom electrogoniometer within the instrumented linkage. Ankle motion was recorded as the number of degrees of angular movement from that position in either a dorsal or plantar direction. 

#### 2.4.4. Data Reduction and Statistical Analysis

For ankle arthrometry, the data was sampled at 2500 Hz and then groups of 41 numbers were averaged together to create a single data point at approximately 60 Hz, which was then used in the calculations. AP displacement at ±100 N and I-E rotation at ±4 N·m torque, as well as the AP and I-E stiffness divided into low- and high- loading ranges were used as dependent variables. To quantify the elasticity of the ankle complex, secant stiffness was calculated as the change in applied force divided by the resulting change in AP displacement and I-E rotation over a load range [21]. To measure AP stiffness, the data were examined over a low-loading range (±0 to 50 N) and high-loading range (±50 to 100 N) for anterior and posterior motion, respectively. Thus, anterior stiffness and posterior stiffness were defined as force per displacement (N/mm) and calculated by dividing 50 N (load differences between ±0 and 50 N and ±50 and 100 N) by displacement for the respective loads. To measure I-E stiffness, the data were plotted over a low-loading range (±0 to 2 N·m) and high-loading range (±2 to 4 N·m) for inversion and eversion rotation, respectively. Thus, inversion stiffness and eversion stiffness (N·m per degree ROM) were defined as torque (N·m) per degree of range-of-motion (ROM) and calculated by dividing 2 N·m of torque (torque differences between ±0 and 2 N·m and ±2 and 4 N·m) by degrees ROM for the respective torque loads [21,23]. 

Separate one-way repeated-measures analyses of variance (ANOVA) were conducted to compare differences for PF and DF ROM and AP and I-E motion and stiffness across the three support conditions (KT [Kinesio tape], ST [sham-Kinesio tape], and NT [no tape]). Post hoc comparisons were assessed using Fisher’s Least Significant Difference (LSD) Test. 

Time-to-stabilization (TTS) was calculated from the vertical ground reaction force (GRFv) component of the ground reaction force [24]. Two windows of the last 10 s of time-to-stabilization for each trial were analyzed, and the window with the smallest absolute GRF range was accepted as the optimal range-variation value. This value represents the window in which the dancer displayed optimal balance. The vertical data component was identified and starting at the peak GRF, an unbounded third-order polynomial was fitted to the GRFv component. The TTS was determined as the point at which the unbounded third-order polynomial transects the static horizontal line [25,26]. Separate one-way repeated-measures analyses of variance (ANOVA) were conducted to compare differences for PF and DF ROM, and AP and I-E motion and stiffness across the three support conditions (KT [Kinesio tape], ST [sham-Kinesio tape], and NT [no tape]). Repeated measures ANOVAs were conducted to evaluate the effect of support condition (KT [Kinesio tape], ST [sham-Kinesio tape], and NT [no tape]) on TTS by type of jump (sauté arabesque, sissonne ouverte de côté, and sissonne ouverte an evant). Post hoc pairwise analyses were performed using Fisher’s Least Significant Difference (LSD) Test. A level of significance (α) was set a priori at 0.05. All statistics were computed using SPSS statistical software (version 23.0; SPSS Inc., Chicago, IL).

## 3. Results

### 3.1. Ankle Complex Stability

Table 1 shows the results of the analysis of ankle complex stability by support condition. Significant main effects for support were observed for inversion rotation (*F*(2,22) = 12.81, *p* < 0.001; η^2^ = 0.54) and eversion rotation (*F*(2, 22) = 5.301, *p* = 0.013; η^2^ = 0.325), but not anterior displacement (*F*(2, 22) = 0.894, *p* = 0.424; η^2^ = 0.075) or posterior displacement (*F*(2, 22) = 1.72, *p* = 0.202; η^2^ = 0.135). Pairwise comparisons showed Kinesio tape (KT) support significantly restricted inversion rotation compared to NT (*p* = 0.002) and ST (*p* < 0.001). In addition, the KT support significantly restricted eversion rotation compared to NT (*p* = 0.007) and ST (*p* = 0.05). However, no significant differences between ST and NT were found for either inversion rotation (*p* = 0.072) or eversion rotation (*p* = 0.239).

Table 2 shows the results of the analysis of ankle complex stiffness by support condition. For inversion stiffness in the low-load range, we noted a main effect for support (*F*(2,22) = 13.897, *p* < 0.001; η^2^ = 0.558). Kinesio tape (KT) support significantly increased stiffness compared to NT (*p* = 0.016) and ST (*p* = 0.014). In addition, ST significantly increased inversion stiffness compared to NT (*p* = 0.008). No significant support main effects (*p* > 0.05) were found for high-load inversion stiffness or for low- or high-load eversion, anterior, or posterior stiffness ranges (*p* > 0.05). 

### 3.2. Ankle Joint Range of Motion

No statistically significant main effects for support for either PF ROM (*F*(2,22) = 2.947, *p* = 0.073; η^2^ = 0.211) or DF ROM (*F*(2,22) = 1.090, *p* = 0.354; η^2^ = 0.09) were found. Plantarflexion ROM differences among ankle support conditions ranged from 0.5° to 1.74° (KT = 46.91 ± 5.8°; ST = 47.38 ±4.8°; and NT = 48.65 ± 4.6°). Dorsiflexion ROM differences among ankle support conditions ranged from 0.62° to 1° (KT = 26.00 ± 7.1°; ST = 26.62 ± 6.2°; and NT = 27.07 ± 7.5°). 

### 3.3. Time to Stabilization

The results of the ANOVA indicated a significant support condition effect on TTS for JA, sauté arabesque, *F*(2,70) = 3.687, *p* = 0.03, η2 = 0.095. Follow-up tests to evaluate pairwise differences indicated that mean TTS for the ST condition (0.555 ± 0.14) was significantly lower (*p* = 0.02) than the NT condition (0.651 ± 0.205). However, the KT condition (0.583 ± 0.126) was not significantly different (*p* > 0.05) from the ST condition or NT condition. No significant support condition effects on TTS were found for JF, sissonne ouverte an evant, *F*(2,70) = 0.049, *p* = 0.95, η2 = 0.001 or JS, sissonne ouverte de côté, *F*(2,70) = 0.215, *p* = 0.81, η2 = 0.006. The means and standard deviations for the three ballet jump landings by support condition are reported in Table 3.

## 4. Discussion

Despite the high prevalence of ankle injuries in ballet [27,28,29], few studies have investigated the effects of Kinesio taping on the mechanical characteristics of the ankle complex in ballet dancers [10]. Our primary finding based on arthrometric measurement indicates Kinesio taping with tension (KT) compared to ST and NT restricted inversion motion without reducing plantarflexion motion. This finding would be beneficial to the dancer to achieve full pointe while limiting excessive frontal plane motion. The KT support produced approximately 20% greater restriction in inversion-eversion motion and for the ballet dancer, this finding appears beneficial to preventing a lateral ankle sprain. When a ballet dancer is transitioning from flat-footed standing to standing en pointe, there is instability in the frontal plane. Instead of moving directly into plantarflexion, a ballet dancer may invert her foot during the transition, potentially contributing to the mechanism that causes a lateral ankle sprain. Application of Kinesio tape could help restrict inversion-eversion motion without restricting PF and DF ROM. 

We found anterior-posterior translation of the ankle complex was not affected by the Kinesio tape application. This finding supports similar findings reported by Fayson et al. who employed a Kinesio tape configuration designed to restrict anterior displacement [10]. The Kinesio tape application used by Fayson et al. included a strip of tape applied transversely across the anterior ankle to restrict anterior motion. The Kinesio tape application we applied did not include a strip of tape applied in this manner. Our Kinesio tape configuration utilized strips of tape positioned to lock the subtalar joint which may explain why the Kinesio taping we applied effectively restricted inversion rotation. 

To provide an assessment of the tape and supporting ankle complex tissue elasticity (stiffness), load-displacement data were examined over low-load and high-load ranges [21,23]. Although stiffness characteristics and the soft tissue loading response of ankle complex are documented in the literature [19,30,31], the stiffness characteristics and loading response using Kinesio tape are less understood [10]. The KT support significantly increased ankle complex inversion stiffness compared to NT and ST in the low-loading range but not the high-loading range. The ST support also significantly increased ankle complex inversion stiffness in the low-loading range compared to NT. The contribution of the KT and ST supports to ankle complex stiffness in the low-loading range was high. However, towards the extremes of motion (high-load range), the ankle complex soft tissues generally become stiffer as the tissue is loaded that may have diminished the relative contribution of KT support to the total stiffness in this region. In addition, the ST was found to significantly increase inversion stiffness compared to NT, which indicates that the Kinesio tape may have activated subcutaneous mechanoreceptors to improve joint proprioception and stiffness [11]. The stretching effects of the Kinesio tape on the skin in the KT trial are believed to stimulate cutaneous mechanoreceptors, which in turn convey information about joint position and movement. This effect likely induces a tensile/stretching mechanism that increases mechanoreceptor activity through biofeedback mechanisms [32]. Although Kinesio tape applied to the skin without tension (ST trial) likely would not activate mechanoreceptors, when ankle-complex movement was initiated during arthrometric loading, it was possible that stretching of the tape on the skin could have occurred that created cutaneous stimulation that resulted in unexpected kinesthetic feedback. Additional studies are needed to determine the effects of Kinesio tape on proprioception to support its use over other types of elastic tape in the management or prevention of ankle sprain.

Kinesio taping application did not produce a change in low- or high-loading range stiffness of the ankle complex for eversion, anterior, and posterior translation. This finding shows that the tape configuration and/or the amount of tension did not provide additional restraint and indicates the KT and ST provided no additional support to the ankle complex passive constraint structures (joint capsule, bone geometry, ligaments, etc.) [21,23]. In contrast, Fayson et al. found anterior stiffness significantly increased after KT application, despite no change in anterior laxity [10]. These results indicate that Kinesio tape may improve static restraint in the ankle joint without altering peak motion. The inconsistent findings between studies may be attributable to methodological differences and variations in tape application procedures, the nature of skin preparation, the amount of tension generated within tape strips as they are adhered to the skin, and the orientation of tape strips in relation to anatomic structures. Additionally, the modest sample size in the present study (N = 12) may have played a role in limiting the sensitivity of some of the statistical comparisons conducted because of the large effect size required to reach adequate power.

Kinesio tape application with tension did not improve time-to-stabilization (TTS) when compared to ST and no-tape. Previous research using TTS as a measure of dynamic postural control used non-dancers who performed various jumping and hopping tasks that ended in single-leg landing on a force plate [10,26]. Participants in the study by Fayson et al. [10] performed a forward hop, lateral hop, medial hop, and backward hop, causing them to transition from a dynamic to a static state and reported the Kinesio tape application did not have an effect on dynamic postural control. The present study employed common ballet jumps that required the dancer to jump forward and laterally with the non-weight bearing leg either in a position of abduction or extended posteriorly. Jump landing with the non-weight bearing leg positioned away from the body’s midline required eccentric strength, coordination, and stability of the ankle joint when landing which likely created different joint and neuromuscular control demands similar to those previously reported. Although the current study found that Kinesio taping with tension restricted ankle inversion, the effect of limiting ankle inversion on altered ballet landing mechanics at the knee and hip joints is unknown. Previous research has shown that ankle taping using standard athletic tape reduced forces at the more proximal knee joint during various dynamic sporting maneuvers [33]. Future research into the neuromechanical effects of taping on the lower-limb function is required to enhance our understanding of the mechanisms behind ankle taping.

Wikstrom et al. [34] investigated dynamic postural stability in subjects with braced and functionally unstable ankles. While they did not find improvement in dynamic postural stability, they reported that the vertical stability index was significantly lower for the braced conditions compared with the control condition. The authors suggested that external ankle support may aid in attenuating vertical ground reaction forces. Since no effect on TTS was found in the present study, Kinesio tape application with tension did not affect the vertical ground reaction forces and time-to-stabilization following the jumping tasks. Kinesio tape has significantly less mass than traditional taping and bracing and is primarily elastic in nature, which may detract from its ability to supply structural support to reduce or affect vertical ground reaction forces. The lack of significant findings in TTS with KT support may also be attributed to the dancers utilizing other neuromuscular balance strategies, such as a hip strategy to maintain postural control. A limitation of this study is that the dancers who participated were healthy and had not experienced an ankle injury within the six months prior to testing. Thus, they may not have demonstrated decreased neuromuscular control during their jump landing task that is oftentimes associated with functional instability after an ankle sprain injury. 

## 5. Conclusions

Female ballet dancers who perform en pointe dance in extreme ranges of motion on a very small, potentially unstable base. The extreme positions require balance control and dependence on the ankle complex for support. Since ballet dancers do not wear traditional taping and bracing due to motion restrictions and unappealing aesthetic nature, this investigation of alternative support was performed. 

Our overall findings indicate that application of Kinesio tape with or without tension did not improve time to stabilization in ballet dancers when landing from a jump. Since impaired dynamic balance is a risk factor for ankle sprain, future research using individuals with previous ankle injury may be necessary to assess the effect of Kinesio taping on time to stabilization. For ankle complex stability, the results demonstrate that Kinesio taping with tension significantly restricted inversion-eversion rotation and increased inversion stiffness. These findings indicate that Kinesio taping with tension restricted frontal plane motion of the ankle complex without reducing plantar- and dorsi-flexion range of motion. 

The present study revealed that the method of Kinesio tape application affects the extent to which talocrural-subtalar joint motion is restrained. Protection of the ankle ligaments is particularly important when helping the ballet dancer safely return to participation after sustaining an ankle sprain injury. Additional research using individuals with a history of previous ankle sprain is warranted to determine the effects of different Kinesio taping configurations on ankle complex motion and TTS with the goal to reduce the high rate of lateral ankle sprains experienced by the ballet dancer.

## Figures and Tables

**Table 1:** Arthrometric Measurements of Passive Ankle Complex Motion by Support Condition.

Support Condition	Anterior Displacement (mm)	Posterior Displacement (mm)	Inversion (° ROM)	Eversion (° ROM)
Kinesio Tape	10.00 ± 3.3	10.36 ± 3.2	28.17 ± 6.7 ^a^	23.37 ± 4.9 ^b^
No Tape	12.12 ± 3.5	11.64 ± 3.4	35.96 ± 9.1	28.14 ± 8.3
Sham-KT	11.08 ± 3.4	11.48 ± 2.3	32.68 ± 5.7	26.05 ± 5.5

Abbreviations: mm, millimeter; ° ROM, degrees range of motion. ^a,b^ Kinesio Tape significantly restricted inversion and eversion rotation compared to No Tape and Sham-KT (*p* < 0.05).

**Table 2:** Arthrometric Measurements of Ankle Complex Stiffness in Low-Load Range and High-Load Range by Support Condition.

	Anterior (N·mm)		Posterior (N·mm)		Inversion (N·M/°)		Eversion (N·M/°)	
	Low Load Range	High Load Range	Low Load Range	High Load Range	Low Load Range	High Load Range	Low Load Range	High Load Range
KT	10.91 ± 6.3	10.75 ± 4.7	14.12 ± 8.7	10.39 ± 4.5	0.167 ± 0.07 ^a^	0.157 ± 0.03	0.196 ± 0.08	0.191 ± 0.03
NT	9.03 ± 4.1	9.86 ± 2.5	10.90 ± 3.9	9.30 ± 4.7	0.102 ± 0.04	0.180 ± 0.05	0.165 ± 0.11	0.187 ± 0.04
ST	10.21 ± 4.2	10.82 ± 5.9	11.31 ± 3.6	8.71 ± 3.3	0.111 ± 0.04 ^b^	0.175 ± 0.02	0.176 ± 0.08	0.183 ± 0.04

Abbreviations: KT, Kinesio tape; NT, No tape; ST, Sham-Kinesio tape; N·mm, Newton·millimeter; N·M/°, Newton·meter per degree. ^a^ KT significantly greater than NT and ST (*p* < 0.05). ^b^ ST significantly greater than NT (*p* = 0.008).

**Table 3:** Time-to-stabilization (M ± SD) by Support Condition and Type of Ballet Jump.

Support Condition	Jump Type	Time-to-Stabilization(s)
Kinesio Tape No Tape Sham-Kinesio Tape	JA	0.583 ± 0.13
	JA	0.651 ± 0.21
	JA	0.555 ± 0.15
Kinesio Tape No Tape Sham-Kinesio Tape	JF	0.626 ± 0.13
	JF	0.628 ± 0.12
	JF	0.634 ± 0.09
Kinesio Tape No Tape Sham-Kinesio Tape	JS	0.585 ± 0.13
	JS	0.592 ± 0.12
	JS	0.617 ± 0.33

Abbreviations: JA, sauté arabesque; JF, sissonne ouverte an evant; JS, sissonne ouverte de côté.
